# Factors Associated with Mental Health Problems among Malaysian Children: A Large Population-Based Study

**DOI:** 10.3390/children8020119

**Published:** 2021-02-07

**Authors:** Norhafizah Sahril, Noor Ani Ahmad, Idayu Badilla Idris, Rajini Sooryanarayana, Mohamad Aznuddin Abd Razak

**Affiliations:** 1 Institute for Public Health, National Institutes of Health, Ministry of Health Malaysia, Setia Alam, Shah Alam 40170, Malaysia; drnoorani@moh.gov.my (N.A.A.); aznuddin.ar@moh.gov.my (M.A.A.R.); 2 Department of Community Health, Faculty of Medicine, University Kebangsaan Malaysia Medical Centre, Kuala Lumpur 56000, Malaysia; idayubadilla.idris@ukm.edu.my; 3 Family Health Development Division, Ministry of Health Malaysia, Putrajaya 62590, Malaysia; drrajini@moh.gov.my

**Keywords:** mental health, children, National Health and Morbidity Survey (NHMS)

## Abstract

Mental health problems are a major public health issue, particularly among children. They impair children’s development, academic achievement, and ability to live a productive life. The present study aimed to determine the prevalence and factors associated with mental health problems among children aged 5 to 15 years old in Malaysia. Data from the National Health and Morbidity Survey (NHMS) 2015 were analyzed. A validated Strengths and Difficulties Questionnaire (SDQ) was used. The overall prevalence of mental health problems among children in Malaysia was 11.1%. Multiple logistic regression analysis revealed that for every year increment in age, mental health problems decreased by 5%. Further analysis found that children who had fathers with a non-formal education and worked in the private sector, had parents who were widowed or divorced, and had either parent with mental health problems were more likely to have mental health problems themselves. Children from the lower socioeconomic group and who had either parent with mental health problems had higher odds of having mental health problems in Malaysia.

## 1. Background

Good mental health in children means reaching age-appropriate developmental and emotional milestones, and at the same time allowing them to learn healthy social skills and how to cope when problems are encountered. Mentally healthy children have good quality of life and can function well at home, in school, and in their communities [1]. Mental health problems affect a significant number of children worldwide. The most common children’s mental health problems include depression, anxiety, attention deficit hyperactivity disorder (ADHD), behavior disorders, and conduct disorders [2]. Mental health problems may severely influence children’s development, their educational attainments, and their potential to live fulfilling and productive lives [1].

The reported prevalence of mental health problems among children is 10–20% globally [3]. A meta-analysis of 41 studies conducted between 1985 and 2012 in 27 countries showed that the prevalence of mental disorders in children was 13.4% (CI 95% 11.3–15.9) [4]. Other epidemiological studies reported by Barkmann and Schulte-Markwort [5], using meta-analysis based on 33 German epidemiological studies, found that the prevalence of mental health problems among children was 17.6%. The BELLA study during the German National Health Interview and Examination Survey among Children and Adolescents reported that the prevalence of mental health problems was 9.8% [6]. In addition, three major surveys of the mental health of children and young people in England were carried out in 1999, 2004, 2014, and 2017. Based on the Mental Health of Children and Young People Survey (MHCYP), the prevalence of mental health problems in the United Kingdom among 5- to 15-year-old children increased from 9.7% in 1999 and 10.1% in 2004 to 11.2% in 2017. Subsequently, in 2017, one in eight (12.8%) children were recorded to have mental health problems [7].

In Asian countries, the prevalence of mental health problems among children and adolescents varied. The prevalence of mental health problems among 2679 children aged 10–15 years old from 25 provinces in China was 20.3%, based on the Centre for Epidemiologic Studies Depression Scale (CES-D) rating [8]. In India, the results of a meta-analysis of epidemiological studies from India showed that the prevalence of mental health problems among children was 7% in the community and 23% in schools [9,10]. Adding to this body of knowledge, the prevalence of mental health problems among children in Vietnam ranged from 8% to 29%, with varying rates across different provinces and genders [11]. Another epidemiological survey of a nationally representative population from 10 out of 63 provinces in Vietnam recorded that the overall prevalence of children’s mental health problems was 12.0% [12]. In Bangladesh, the prevalence of mental health problems was 14.6% (95% CI 11.4, 17.9) among children aged 2–9 years that were identified during a population-based survey involving 2231 households [13]. The overall prevalence of mental health problems among children 6–18 years old in Iran was 26.0% [14]. Mental health problems that were reported in this survey included conduct disorders (34.7%), which was the most common problem in children, followed by peer-relationship problems (25.4%), emotional problems (24.5%), hyperactivity (23%), and impairment of prosocial behavior (5.7%). Another study conducted in Malaysia found that the prevalence of mental health problems in Malaysian school children was 8.5% when reported by parents [15].

During the National Health and Morbidity Survey (NHMS) conducted in Malaysia, it was revealed that there was an increasing trend of mental health problems among children aged 5–15 years from surveys conducted in 1996 (13.0%), 2006 (19.4%), and 2011 (20.0%). In 1996, the prevalence of mental health problems among children was reported higher among males, other ethnic backgrounds (excluding Bumiputras, who are the main ethnic group in this country), children who resided in rural areas, children with parents who have no formal education, and unmarried parents. Results in 2006 also showed that the prevalence of mental health problems in children was higher among males and rural dwellers. In addition, children of Indian ethnicity, children of parents with a secondary education, and children with married parents were also reported to have a higher prevalence of mental problems. The results of the 2011 survey showed that male children and adolescents from less-affluent families were significantly associated with mental health problems [16].

Other Western research has recorded other factors that were associated with mental health problems among children. These factors include female gender, parental unemployment [17,18], stressful life events [19,20], and parental stressors in daily life [10]. Furthermore, children whose parents who had chronic health problems [19,21] have also been found to develop mental health problem. Changes in family environment may also contribute to mental health problems in children, such as increased rates of single parents [22], family conflicts [23], and parental mental health problems [24,25,26]. 

The structure of mental health in children and adolescents was characterized by many studies into internalized and externalized problems [27]. Internalized problems appear in the form of withdrawal, somatic complaints, anxiety, fearfulness, and depression [28]. Studies found that internalized problems tended to increase with age [29,30]. In addition, girls show higher increase in internalizing problems across time [29]. On the other hand, externalized problems take the form of hyperactivity, aggression, defiance, and structure behavior. A majority of children with moderate or high levels of externalized issues in early in development showed a reduction in behavioral problems after preschool years [31]. Several risk factors such as family adversity, maternal depression, low socioeconomic status, and single-parent status were found to be among the strongest predictors of later externalized problems [32,33,34]. The SDQ was typically used to investigate internalization/externalization disorders. A recent study using SDQ among Australian children aged 7–17 years found that the prevalence of internalized and externalized problems was 18.0% and 11.3%, respectively. Internalized and externalized problems were associated with Aboriginal status, age, and gender. Aboriginal children scored higher across all component subscales (emotional symptoms (*p* < 0.01), peer relationship problems (*p* < 0.0001), conduct problems (*p* < 0.0001), and hyperactivity (*p* < 0.0001)) [35].

The primary objective of the nationwide population-based survey that was carried out in 2015 in Malaysia was to determine the prevalence of mental health problems among children, and at the same time, to examine the possible risk factors that may contribute to those mental health problems. To our knowledge, this was the first nationwide study in Malaysia that looked into this aspect.

## 2. Methodology

### 2.1. Sample and Design

The National Health and Morbidity Survey (NHMS) 2015 was conducted by the Institute for Public Health, Ministry of Health Malaysia to provide health-related community-based data and information to support the Ministry of Health Malaysia in reviewing its health priorities, program strategies, activities, and planning of its allocation of resources. Geographically, NHMS 2015 covered both urban and rural areas for every state in Malaysia. The target population was residence in the non-institutional living quarters (LQs). Selection of samples was conducted by the Department of Statistics Malaysia using the updated 2014 sampling frame. Based on the frame, the geographical areas in Malaysia were divided into enumeration blocks (EBs). There were about 75,000 EBs in Malaysia, and each EB comprised 80 to 120 LQ with an average population of 500 to 600 people. In this survey, the sample size was calculated based on a single proportion formula for prevalence estimation. As this survey consisted of several topics, the sample size for the whole survey was based on the biggest sample size. The allocation of samples to the states, urban and rural, was done proportionally to the population size. NHMS 2015 employed a two-stage stratified random sampling design to ensure national representativeness. The two strata were the primary stratum, which was made up of states in Malaysia, including federal territories, and the second stratum, which was made up of urban and rural strata formed within the primary stratum. Sampling involved two stages: the primary sampling unit (PSU), which was for enumeration blocks (EBs), and the second sampling unit (SSU), which was for living quarters (LQs) within the selected EB. In this survey, out of 10,428 EBs, 536 EBs were selected from urban areas, with the remaining 333 EBs from rural areas, based on the proportion of Malaysian residents. In each EB, a total of 12 LQs were randomly selected, and all households and individuals within the selected LQs were invited to join this survey. The selection of EBs and LQs were done by the Department of Statistics Malaysia randomly. Based on the list of the occupied LQs, data collection teams made an appointment to visit for the interview. A minimum of three visits were made for each LQ. Consented individuals were invited to join the survey and respond to the questionnaires in NHMS 2015, including a mental health questionnaire. Prior to data collection, a training course was conducted for the teams, which consisted of field supervisors, team leaders, nurses, and interviewers. Data collection were done from March to June 2015 [36].

One of the objectives of this survey was to assess the prevalence of mental health among children residing in randomly selected living quarters in Malaysia. This study adopted the use of a secondary data analysis from the NHMS 2015 [36]. A total of 5182 (out of 5832) children aged 5 to 15 years responded to questionnaire, resulting in a response rate of 88.9%. Of those who responded, only 4309 (83.2%) had all information (completed data) for both child and parents. For the purposes of this study, a representative sample of 4309 5- to 15-year-old children was analyzed. Parents or guardians were invited to answer the questions related to mental health problems on behalf of their children over the last six months using the SDQ, a validated self-administered questionnaire that was in either Malay or English [37,38]. There were certain modules such as alcohol, health literacy, mental health (adult) and mental health (children) that were meant to be answered by the respondents or parents alone. Those respondents who had communication problems such as cognitive impairment, low literacy, or a language barrier could not be assisted by the interviewer, since the questionnaire was self-administered. Consent was obtained from the parents or guardians prior to the data-collection process. All survey methods were reviewed and approved by the Medical Research and Ethics Committee, Ministry of Health Malaysia prior to implementation. Details of the sampling strategy can be found in [36]. All subjects gave their informed consent for inclusion before they participated in the study. This study was conducted in accordance with the Declaration of Helsinki 1975, as revised in 2013. The protocol was approved by the Ethics Committee of Medical Research and Ethics Committee, Ministry of Health Malaysia (NMRR-14-1064-21877).

### 2.2. Study Instrument

Mental health problems among children were determined using the Strengths and Difficulties Questionnaire (SDQ). The Malay version of the SDQ has acceptable internal consistency, with a Cronbach’s alpha coefficient of 0.67 based on parent ratings of a large group of children in Malaysia [37]. In this study, The SDQ was completed by the parents or caregivers of the respective children. For example, parents were asked whether their children were considerate of other people’s feelings. The available responses were: “Not True,” “Somewhat True,” or “Certainly True.” The SDQ is a 25-item instrument comprising five domains, with each domain comprising five items. These domains are: Total Difficulties Score, Emotional Problems, Conduct Problems, Hyperactivity Problems, Peer Problems, and Pro-Social Skills. Each item in the questionnaires were answered based on a Likert scale of Not True, Somewhat True, and Certainly True. For each of the mentioned domains, the score could be scaled up to pro-rata if at least three items were completed. Each domain score (i.e., Emotional, Conduct, Hyperactivity, Peer and Pro-Social) had a score range of 0–10. The results score ranged from 0 to 40, and was counted as missing if one of the four components score was missing. Total Difficulties were generated by summing scores from all the scales except the Pro-Social scale [39]. Internalizing and externalizing psychopathology can be accessed via the SDQ. The two-subscale structure involves the items from the emotional symptoms and peer-relationship problems subscales being combined to form a single “internalizing” subscale (10 questions; possible score range: 0–20), and the items from the conduct problems and hyperactivity/inattention subscales being combined to form a single “externalizing” subscale (10 questions; possible score range: 0–20) [40].

Mental health problems among parents were screened using a locally validated GHQ-12. This validated GHQ-12’s sensitivity and specificity were 81.3% and 75.3%, respectively, with a positive predictive value (PPV) of 62.9%, as well as an area under the receiver operating characteristic (ROC) curve of more than 0.7. The Cronbach’s alpha value of the GHQ-12 was 0.85 [41]. The GHQ-12 is the shorter version of the original GHQ-60, which was designed as a self-administered screening test for detecting minor psychiatric disorders among respondents in community settings [31]. The GHQ-12 has a total of 12 questions, and each question has four different responses. For example, question one was “Have you recently been able to concentrate on whatever you are doing?”. The available responses were: “better than usual,” “same as usual,” “less than usual,” or “much more than usual.” For all 12 questions, the first two responses were considered as positive and given a score of 0, while the remaining two options were considered as negative and scored as 1. In this study, parents were considered to have mental health problems if the total score of their GHQ-12 was 3 and above [42].

### 2.3. Definition of Variables

The children were considered to have mental health problems if the Total Difficulties score was 14 or more [39], and this served as the dependent variable. The independent variables were age of child, gender, residential area, ethnicity, citizenship, age of father and mother, education of father and mother, occupation of father and mother, parental marital status, and the mental health problems in either parent.

The major ethnic groups in Malaysia were categorized as Malays, Chinese, and Indians, followed by “other Bumiputras” and “others.” The “other Bumiputras” category comprised indigenous groups and local residents of Sabah and Sarawak. The “others” category included foreigners and immigrants (both legal and illegal) who were then residing in Malaysia.

The education level of parents/caregivers was based on the Malaysian education system. Education level referred to the highest level of schooling completed in a public or private institution that provided formal education. Those who had never attended school in any of the educational institutions that provided formal education were considered as having no formal education. Primary education referred to Standard 1 to 6 or equivalent. Those whose highest-level of education was from Form 1 to 5 or equivalent were considered as having a secondary education, while tertiary education referred to those who obtained a diploma or higher qualifications. Household income was classified into three groups: low (less than MYR 4999), middle (MYR 5000–MYR 9999) and high (MYR 10, 000 and above).

### 2.4. Analysis

As the sampling was a multi-stage stratified sampling, the analysis was conducted accordingly to ensure the sample weight and design effect were accounted for during the analysis. Data analyses were conducted using SPSS version 19.0 utilizing complex survey design. The overall prevalence of mental health problems and the prevalence of responses in each domain were determined. Bivariate analysis was done to examine the association between the independent variables and mental health problems. As the dependent variable was dichotomous, we used a logistic regression model to produce crude odds ratios (ORs) as a measure of association. The adjusted ORs, with their respective 95% confidence intervals (CIs), were then calculated. A *p*-value of less than 0.05 was considered statistically significant.

## 3. Results

Table 1 shows the sociodemographic profile of the respondents to the NHMS 2015 study. A total of 4309 children aged 5 to 15 years old in Malaysia participated in this research. The mean age of the children was 9.96 years, with a standard deviation of 3.13. Three-quarters of the respondents resided in urban areas, with almost equal distribution according to gender. In terms of ethnicity, 61.8% of the respondents were Malays, followed by Chinese (17.4%), other Bumiputras (12.6%), Indians (6.1%), and others (2.1%). A majority of the respondents were Malaysian citizens. The mean age of the fathers was 43.74 (SD. 8.24) years, while the mean age of the mothers was 40.47 (SD. 7.91) years. More than half of the fathers and mothers attained a secondary education level. A majority of fathers worked in the private sector, while a majority of mothers were unpaid homemakers. According to marital status, more than 90% of the respondents were married. It was also noted that more than half of the respondents had a low family income (<MYR 4999). About 42.5% of parents were reported to have some form of mental health problems.

With regard to the SDQ subscales, the prevalence of emotional problems among children were 15.3%; peer problems, 31.0%; conduct problems, 15.7%; and hyperactivity problems, 4.7%. The prevalence of internalizing and externalizing problems among children was 19.7% and 4.8%, respectively (Table 2).

The overall prevalence of mental health problems among children and adolescents in this study was 11.1%, which represents 456,142 of the total population of children in Malaysia at the time of the survey. The mean age of the children was 9.63 years, with a standard deviation of 3.032. The prevalence of mental health problems was reported to be higher among female children, those residing in rural areas, other Bumiputras and non-Malaysian citizens. Children whose fathers had no formal education and mothers with a primary education showed a higher prevalence of mental health problems. The prevalence of mental health problems was reported to be higher among children who had both their father and mother working in the private sector. A higher prevalence of mental health problems was also reported among children who had parents who were not married, had a low family income, and who had either parent with mental health problems (Table 3).

Subsequently, in this study, multiple logistic regression analysis revealed predictors of mental health problems among children. Mental health problems decreased by 5% when the age of children increased (aOR: 0.95, *p* = 0.022). In other words, mental health problems were found to be higher among younger children. In addition, Malaysian citizens were reported to have a higher risk of having mental health problems compared to non-Malaysians. Other significant variables that were associated with mental health problems were fathers with a non-formal education (aOR: 4.34, *p* = 0.003) or who worked in the private sector (aOR: 3.60, *p* = 0.044), parents who were widowed or divorced (aOR: 5.11 *p* = 0.047), and either parent having mental health problems (aOR: 1.93, *p* < 0.001) (Table 3).

## 4. Discussion

Mental health surveys that are conducted during large population-based studies serve as an important platform to document the prevalence of mental health problems in the community. These surveys lead to the understanding of the magnitude and extent of such problems, which helps in the planning of mental health services, including for children and adolescents. Mental health services aim to prevent, detect, and subsequently treat children with psychiatric morbidities and promote their normal development, and thus enable these young people to reach their full potential. At the same time, community mental health surveys also are able to identify important factors that are associated with mental health problems so that suitable interventions can be introduced, taking into account the identified risk factors.

The study showed that based on parent-reported SDQs, peer problems and conduct problems were the most frequent difficulties for children. The result of the present study was in agreement with other studies that used the same tool [14,43]. The prevalence of Australian children 7–17 years old that experienced internalized problems was 18.0%, slightly lower than our results (19.7%); while the findings for externalized problems among children in Malaysia were lower (4.8%) compared to their findings (11.3%) [35].

In the present study, the prevalence of mental health problems among children was 11.1%, which was lower when compared to the prevalence globally [3,4]. The prevalence of mental health problems among children reported in Malaysia also appeared to be lower when compared with other Asian countries such as Vietnam (12.0%) [12], Bangladesh (14.6%) [13], China (20.3%) [8], and Iran (26.0%) [14].

The present study demonstrated that older children had lower odds of mental health problems, which was consistent with the study conducted by Amstadter et al. [44]. An increase in age was well documented as a protective factor in surveys using samples of children from the United States [45]. Further analysis by subscales in the present study found that the hyperactivity subscale (aOR: 0.88, *p* < 0.001) and peers subscale (aOR: 0.96, *p* = 0.009) had an association with the age of children. Gender was not found to be significantly associated with mental health problems among children in the current study, a trend which was consistent with results from Vietnam [44]. Our result also revealed no differences by locality, similar to previous studies [16].

A lower education of fathers showed a significant association with child mental health problems in this study. In Spain, parental education was reported be the strongest risk factor for child mental health problems among 4- to 11-year-old children, with an OR of 3.7 (95% CI 2.4–5.8) for the lowest educational level [46]. In the United States, children of fathers who attained less than a high-school education were reported to have increased odds of mental health problems, compared to those with fathers who were high-school graduates [21]. Furthermore, low educational attainment of parents has been shown to be independently associated with reduced utilization of child mental health resources, as well as increased severity and duration of child mental health problems [47,48].

Perna et al. [49] observed that parental occupation has an association with children’s mental health problems. In Spain, mothers’ “homemaker” status was related to children’s mental health [50], while our results showed that fathers working in the private sector were associated with higher odds of mental health problems in their children. Previous studies indicated that the work stress of parents can lead to psychological distress and mental illness among parents [51,52]. The stress that parents bring home from their jobs can affect the atmosphere in the home, and thereby introduce stress into children’s lives [53].

Children of parents who were widowed or divorced had 5.11 times higher odds of having mental health problems compared to children of parents who were married. This result was supported by previous studies that showed that children who were raised by single parents had a higher probability of developing mental health problems compared to children raised in two-parent families [21,49,54,55]. Divorced parents can affect the social, emotional, and cognitive adjustment of children [56]. Children from divorced families were reported to have higher odds of involvement in substance abuse, and had a significantly higher prevalence of psychiatric disorders [57].

In this study, parental mental health problems contributed to a significant risk in the development of mental health problems among their children [7]. Children of parents with mental health problems were found to have mental health problems themselves, i.e., nearly two times more often compared to children whose parents did not have mental health problems. This result was quite similar to that of a study conducted in Germany that found that the risk for children of parents with mental health problems to report mental health problems themselves was almost two times higher than the risk of children of parents without mental health problems [6]. Moreover, a study by McLaughlin et al. [47] reported that the risk of children to develop any psychiatric disorder was 1.8–2.9 times higher if one parent was affected by mental health problems. Another study by Siegenthaler et al. [26] also revealed that children of parents with a severe mental illness had a 50% chance of developing any mental illness, and a 32% chance of developing a severe mental illness. This result not only suggests that the genes that they inherited from their parents may make them more vulnerable to mental illnesses, but also suggests their situation and the environment in which they grew up could be factors [58]. Furthermore, the risk factors for parental mental health problems, such as parental daily strain, parental chronic diseases, or stressful life events, can indirectly serve as risk factors for mental health problems among their children [6,19,47].

This study also encountered a few limitations. First, the tool used in this study was based on self-reported data by parents, and no clinical assessment was done. Second, this was a cross-sectional design study, which thereby limited the determination of the temporal relationship between the studied independent variables and mental health problems, in order to establish a true cause–effect relationship. However, it should be noted that this study had a large amount of nationally representative sample data that were representative of the population of children in Malaysia.

## 5. Conclusions

A younger age, single parents, parents working in the private sector, lower parental education, and parental mental health problems were major factors associated with mental health problems among children in Malaysia. Consequently, there is an urgent need for the implementation of targeted prevention and treatment strategies specifically designed for the risk group of children with mental health problems. The training of health care workers and primary care providers should be strengthened and take into account development considerations of identity, emotional, social, cognitive, and biological bases to enable them to conduct opportunistic screening in order to detect risk factors and early signs of mental health problems among children, so that they can be referred early for specific interventions. Further research should be carried out to monitor the prevalence and trends of mental health problems among children in Malaysia.

## Figures and Tables

**Table 1:** Sociodemographic profile of the respondents (*n* = 4309).

	Unweighted Count	Percentage (%)
**Age of Children(year) ^a^**	9.96	3.132
**Gender**		
Male	2138	51.8
Female	2171	48.2
**Locality**		
Urban	2513	75.9
Rural	1796	24.1
**Ethnicity**		
Malay	3047	61.8
Chinese	464	17.4
Indian	289	6.1
Other Bumiputras	407	12.6
Others	102	2.1
**Citizenship**		
Malaysian	4229	97.8
Non-Malaysian	80	2.2
**Age of Father(year) ^a^**	43.74	8.242
**Age of Mother(year) ^a^**	40.47	7.910
**Father’s Education**		
Non-formal	39	1.0
Primary	503	15.2
Secondary	1837	52.4
Tertiary	948	31.3
**Mother’s Education**		
Non-formal	90	2.0
Primary	622	16.1
Secondary	2296	55.4
Tertiary	1016	26.5
**Father’s Occupation**		
Government/Semi Government	686	19.1
Private employee	1399	48.8
Self-employed	1061	29.1
Unpaid worker/homemaker	17	0.6
Retiree	82	2.4
**Mother’s Occupation**		
Government/Semi Government	660	14.8
Private employee	821	24.2
Self-employed	622	15.5
Unpaid worker/homemaker	1672	44.9
Retiree	21	0.6
**Marital Status of parents**		
Never married	53	1.2
Married	4029	94.0
Widowed/Divorced	220	4.7
**House Income**		
Low	3086	67.7
Middle	920	24.0
High	296	8.3
**Either parent has mental health problems**		
No	2476	57.5
Yes	1833	42.5

^a^ Mean (SD).

**Table 2:** Prevalence of mental health problems among children by subscale (*n* = 4309).

Subscale	Prevalence (95% CI)	Subscale	Prevalence (95% CI)
Emotional Problems	15.3 (13.76,16.96)	Internalizing Problems	19.7 (17.93, 21.52)
Peer Problems	31.0 (28.93,33.16)		
Conduct Problems	15.7 (14.22,17.29)	Externalizing Problems	4.8 (3.98, 5.79)
Hyperactivity Problems	4.7 (3.86,5.69)		

**Table 3:** Factors associated with mental health problems among children in Malaysia (*n* = 4309).

	Prevalence (95% CI)	Crude OR (95% CI)	*p*-Value	Adjusted OR (95% CI)	*p*-Value
**Age of Children(year)**	9.63 (3.032) ^a^	0.96 (0.93, 0.99)	0.014	0.95 (0.92, 0.99)	0.022
**Gender**					
Male	11.0 (9.44, 12.86)	1		1	
Female	11.3 (9.66, 13.11)	0.87 (0.72, 1.05)	0.151	0.80 (0.63, 1.02)	0.076
**Locality**					
Urban	10.8 (9.38, 12.45)	1		1	
Rural	12.2 (10.01, 14.70)	1.07 (0.89, 1.30)	0.460	1.06 (0.82, 1.38)	0.649
**Ethnicity**					
Malay	10.1 (8.72, 11.62)	1		1	
Chinese	10.7 (7.60, 14.97)	1.01 (0.74,1.39)	0.937	1.23 (0.81, 1.86)	0.333
Indian	13.4 (8.84, 19.82)	0.93 (0.63,1.39)	0.735	0.75 (0.42, 1.35)	0.340
Other Bumiputras	15.8 (11.41, 21.50)	1.62 (1.22,2.17)	0.001	0.92 (0.60, 1.42)	0.716
Others	11.5 (6.00, 20.90)	1.59 (0.92,2.75)	0.095	2.33 (0.84, 6.50)	0.106
**Age of Father(year)**	42.81 (8.466) ^a^	0.98 (0.97, 1.00)	0.023	0.99 (0.96, 1.01)	0.224
**Age of Mother(year)**	40.32 (8.029) ^a^	0.99 (0.98, 1.01)	0.657	1.01 (0.98, 1.04)	0.448
**Father’s Education**					
Non-formal	19.7 (6.09, 48.27)	2.81 (1.25, 6.33)	0.013	4.34 (1.64, 11.51)	0.003
Primary	14.7 (10.19, 20.62)	1.59 (1.13, 2.24)	0.008	1.54 (0.98, 2.44)	0.063
Secondary	10.7 (8.87, 12.79)	1.31 (1.00, 1.71)	0.050	1.19 (0.85, 1.67)	0.300
Tertiary	8.4 (6.23, 11.11)	1		1	
**Mother’s Education**					
Non-formal	12.6 (6.74, 22.18)	1.52 (0.82, 2.84)	0.184	2.17 (0.82, 5.76)	0.120
Primary	14.2 (10.73, 18.61)	1.55 (1.14, 2.09)	0.005	1.40 (0.87, 2.26)	0.169
Secondary	10.8 (9.15, 12.79)	1.13 (0.89, 1.44)	0.325	1.15 (0.80, 1.65)	0.458
Tertiary	9.7 (7.58, 12.35)	1		1	
**Father’s Occupation**					
Government/Semi-Government	9.1 (6.51, 12.47)	2.12 (0.75, 5.97)	0.156	3.24 (0.92, 11.38)	0.067
Private employee	11.2 (9.07, 13.80)	2.56 (0.92, 7.08)	0.071	3.60 (1.04, 12.48)	0.044
Self-employed	11.0 (8.61, 14.03)	2.37 (0.85, 6.60)	0.098	3.10 (0.90, 10.71)	0.074
Unpaid worker/homemaker	10.6 (2.86, 32.35)	2.60 (0.44, 15.50)	0.294	5.60 (0.73, 42.70)	0.097
Retiree	3.5 (1.21, 9.94)	1		1	
**Mother’s Occupation**					
Government/Semi-Government	8.7 (6.05, 12.33)	1		1	
Private employee	13.7 (10.63, 17.43)	1.32 (0.96, 1.82)	0.087	0.88 (0.58, 1.34)	0.558
Self-employed	10.7 (7.65, 14.75)	1.05 (0.74, 1.49)	0.780	0.84 (0.52, 1.35)	0.473
Unpaid worker/homemaker	10.3 (8.48, 12.45)	0.98 (0.73, 1.31)	0.882	0.72 (0.49, 1.08)	0.110
Retiree	13.3 (3.43, 39.67)	1.98 (0.65, 6.05)	0.231	1.94 (0.21, 18.36)	0.563
**Marital Status of parents**					
Never married	26.8 (15.03, 43.21)	2.39 (1.25, 4.58)	0.009	3.28 (0.33, 32.99)	0.314
Married	10.7 (9.47, 12.07)	1		1	
Widowed/Divorced	16.4 (10.18, 25.44)	1.55 (1.07, 2.26)	0.021	5.11 (1.02, 25.59)	0.047
**Income**					
Low	11.9 (10.36, 13.57)	1.87 (1.17, 2.99)	0.008	1.63 (0.90, 2.92)	0.104
Middle	10.2 (7.77, 13.39)	1.65 (1.00, 2.72)	0.050	1.59 (0.91, 2.77)	0.104
High	8.1 (4.64, 13.85)	1		1	
**Either parent has mental health problems**					
No	8.5 (7.11, 10.08)	1		1	
Yes	14.7 (12.51, 17.10)	1.74 (1.44, 2.11)	<0.001	1.93 (1.51, 2.46)	<0.001

^a^ Mean (SD).

## Data Availability

The data used for this study are not publicly available due to reasons of data protection but are available from the Institute for Public Health, Ministry of Health Malaysia upon reasonable request and with permission from the Director General of Health Malaysia.

## Availability of Data and Materials

The datasets generated and analyzed in the current study are not publicly available due to ethical restrictions.

## Abbreviations

**NHMS**: National Health and Morbidity Survey; **SDQ:** Strengths and Difficulties Questionnaire; **ADHD**: attention deficit hyperactivity disorder; **MHCYP**: Mental Health of Children and Young People Survey; **CES-D**: Centre for Epidemiologic Studies Depression Scale.
